# The recovery of North Atlantic right whales, *Eubalaena glacialis*, has been constrained by human-caused mortality

**DOI:** 10.1098/rsos.180892

**Published:** 2018-11-07

**Authors:** Peter Corkeron, Philip Hamilton, John Bannister, Peter Best, Claire Charlton, Karina R. Groch, Ken Findlay, Victoria Rowntree, Els Vermeulen, Richard M. Pace

**Affiliations:** 1 Protected Species Branch, NOAA Northeast Fisheries Science Center, Woods Hole, MA 02543, USA; 2 Anderson Cabot Center for Ocean Life, John H. Prescott Marine Laboratory, New England Aquarium, Boston, MA 02110, USA; 3 The Western Australian Museum, Welshpool DC, Western Australia 6086, Australia; 4 Mammal Research Institute Whale Unit, Department of Zoology and Entomology, University of Pretoria, Hatfield, South Africa; 5 Centre for Marine Science and Technology, Curtin University, Bentley, Western Australia, Australia; 6 Projeto Baleia Franca/Instituto Australis, Imbituba, Santa Catarina 88780-000, Brazil; 7 Research Chair: Oceans Economy, Cape Peninsula University of Technology, Cape Town, South Africa; 8 Department of Biology, University of Utah, Salt Lake City, UT 84112, USA; 9 Instituto de Conservación de Ballenas, Capital Federal, Buenos Aires 5411, Argentina

**Keywords:** population projection model, whale conservation, entanglement mortality, geographical comparison

## Abstract

North Atlantic right whales (NARW), *Eubalaena glacialis*, were nearly exterminated by historical whaling. Their abundance slowly increased up until 2010, to a maximum of fewer than 500 whales, and since then they have been in decline. We assessed the extent to which the relatively slow increase demonstrated by NARW was intrinsic, and how much could be due to anthropogenic impacts. In order to do so, we first compared calf counts of three populations of Southern right whales (SRW), *E. australis*, with that of NARW, over the period 1992–2016. By this index, the annual rate of increase of NARW was approximately one-third of that of SRW. Next we constructed a population projection model for female NARW, using the highest annual survival estimates available from recent mark–resight analysis, and assuming a four-year calving interval. The model results indicated an intrinsic rate of increase of 4% per year, approximately twice that observed, and that adult female mortality is the main factor influencing this rate. Necropsy records demonstrate that anthropogenic mortality is the primary cause of known mortality of NARW. Anthropogenic mortality and morbidity has limited the recovery of NARW, and baseline conditions prior to their recent decline were already jeopardizing NARW recovery.

## 1. Introduction

The near-extinction of several species of baleen whales by commercial whaling removed these animals as functional components of marine ecosystems [1]. Today many populations are increasing in abundance, but some are recovering more slowly than others [2]. Are these differences in recovery due to ongoing anthropogenic impacts or intrinsic ecological factors [3]? Determining the extent to which anthropogenic impacts impede whales' recovery is important. For biodiversity conservation, some whale species (or populations) are at risk of extinction as they number in the dozens [4,5], or in the low hundreds and are declining [6]. As ecosystem service providers, baleen whales can be important ecosystem engineers, for example by provision of iron in systems where it is lacking [7], or by cycling nutrients through the water column [8]. Ensuring whales' recovery will mean that they may resume their prior ecosystem roles, thereby contributing to marine ecosystem integrity. Here we compare patterns of recovery of four populations of two species of right whales, *Eubalaena*, and attempt to assess the extent to which differences in recovery of one are due to anthropogenic activity rather than ecologically intrinsic factors.

There are three species of *Eubalaena*: North Atlantic, *E. glacialis* (NARW); North Pacific, *E. japonica* (NPRW); and Southern, *E. australis* (SRW) right whales. All right whale populations were reduced substantially by historical whaling [2]. There were two populations of NARW, one of which (the eastern) appears to be extinct [9], while the western population off the eastern seaboard of North America has been the subject of substantial research effort throughout their known range since the 1980s [10]. NARW abundance increased between 1990 and 2010 at approximately 2.8% per year, and since then has declined [6]. The species' abundance was estimated at 458 individuals in 2015, using a Bayesian mark–resight analysis of photo-identification data [6]. Of the three species, the habitat of North Atlantic right whales is the most heavily industrialized [10].

Similarly, there are two populations of NPRW; the eastern population of NPRW is believed to number only around 30 individuals [4], and the status of the western population is uncertain, although it appears to be larger than the eastern [11]. There are no records of NPRW births in recent years, and the locality of the calving ground(s) for NPRW remains unknown. Also, there is no time series of NPRW abundance. As there are no data with which NPRW could be compared with other right whales, they are not considered further in this paper.

There appear to be seven populations of SRW, distinguished by the locations of their calving grounds and genetic studies [12]. These are off: the eastern coast of South America (Argentina and Brazil [13]); Tristan da Cunha [14]; the southern coast of Africa (South Africa and Namibia [15,16]); the southwestern and south-central coast of Australia [17]; the southeast and east coast of Australia [18,19]; the Auckland Islands and New Zealand [12]; and the Peruvian and Chilean coasts of western South America [20]. The status of the population that calves around Tristan da Cunha is unknown, as is the extent of immigration between these whales and those observed off either Africa or South America [14,21]. Data collection for the whales off Chile and Peru has been sporadic, and this population is extremely small [20].

Most SRW calve in shallow inshore waters, and calving sites are predictable in time and space. Female right whales with calves tend to remain at the surface and move slowly. This behaviour, coupled with the ease with which right whales can be identified from aerial photographs of their head [6], makes females with calves of the year on the calving grounds relatively easy to survey [22]. This has resulted in long-term survey programmes instituted for those populations where it has been logistically and financially feasible.

Multi-decadal survey data exist for SRW populations off eastern South America [23], southern Africa [24], and southwest Australia [17,25]. An incomplete time series (1995–1998, and the 2006 onwards) exists for SRW calving around the Auckland Islands [26,27]. There are very few data for the SRW populations calving off south-east/east Australia nor for the Peruvian and Chilean coasts of western South America, and no data for the SRW population calving off Tristan da Cunha, so these populations are not considered further.

Here we use calf counts to compare the recovery of NARW with the three populations of SRW for which comparable time-series data are available. To do this, we use the only measure that is directly comparable among populations: a raw minimum count of calves known to be born each year. Further, we question the extent to which the lower rate of increase over time of NARW is intrinsic, or anthropogenically driven. To do this, we construct a matrix population model [28] for female NARW to establish a maximum intrinsic rate of increase for NARW given the conditions in which they currently live, and compare this with the observed rate of increase.

## 2. Methods

### 2.1. Data

We analysed four time-series of counts of calves born each year, from 1992 or 1993 to 2013 or 2016 inclusive. These were: North Atlantic right whales (NARW); SRW off eastern South America (SRWSAm); southern Africa (SRWSAf); and southwest Australia (SRWOz). We did not include the Auckland Islands population as the gap in that time series was too large. We selected 1992 as our starting year as it marked the start of intensive aerial surveys of NARW calving habitat over the winter [29], and comparable datasets are available for the four time series analysed. SRW calve in the austral winter (i.e. mid-year), so for them the calving year is the year in which calving occurs. NARW calve in the boreal winter, with calving starting in December. Here, we follow Pace *et al*. [6] and take December as the starting point for the ‘calving year’, so the 2016 calving year started in December 2016 and ended in March 2017. Details of the survey methods used to collect calf counts over time from each site are provided in electronic supplementary material 1.

### 2.2. Analysis

**Figure 1.**
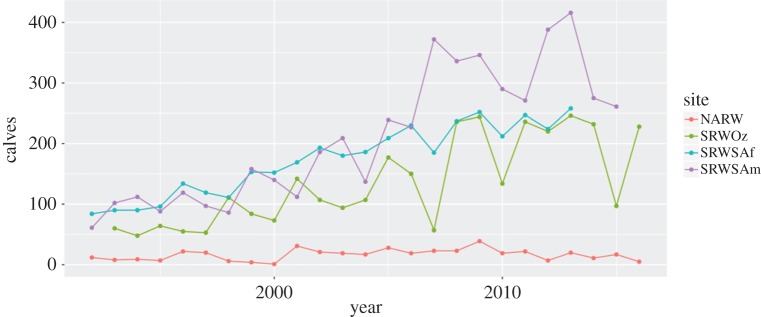
Right whale, *Eubalaena*, calf counts over time, 1992–2016, raw data. NARW: North Atlantic right whale, *E. glacialis*; SRWOz: Southern right whale, *E. australis*, Southwest Australia; SRWSAf: Southern right whale, South Africa; SRWSAm: Southern right whale, eastern South America. R code for the figure is provided in electronic supplementary material 2.

We tested whether the slopes of the relationships of calf counts over time (i.e. average annual increases and their uncertainty) differed between the four populations of right whales under study. Analyses were run in R version 3.4.3 [30], using the libraries *MASS* [31], *ggplot2* [32]; *ggfortify* [33,34]; *sjPlot* [35] and *phia* [36]. Populations were treated as categorical predictors (Sites) with four categories: NARW, SRWOz, SRWSA and SRWSAm. Calf counts were available for Sites over the period 1992–2016 (figure 1), with the exceptions of SRWSA, for which data were from 1992 to 2013, and SRWOz, for which the data were from 1993 to 2016. We tested whether the trend in calf counts over time differed among populations using Generalized Linear Models (GLMs). We ran a Negative Binomial GLM with log-link, including an interaction effect of population and Year. We adjusted *p*-values when making multiple comparisons, using Benjamini & Hochberg's False Discovery Rate [37] (in *phia::testInteractions*). Data and R code are included in electronic supplementary material 2.

### 2.3. Population projection models

**Figure 2.**
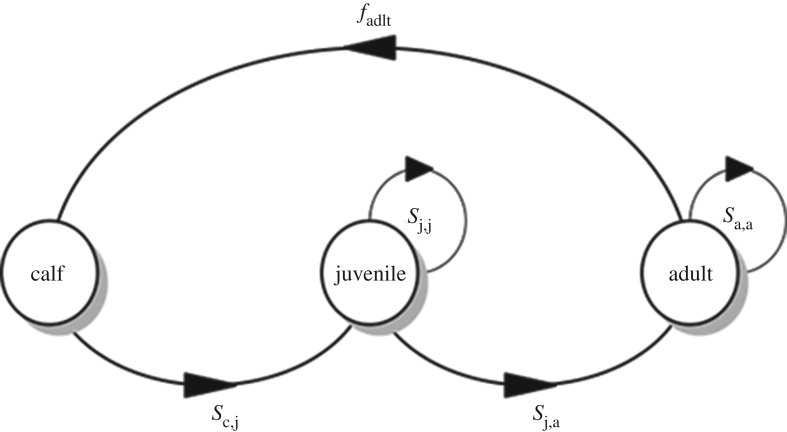
Simplified, stage-structured model of the demography of female North Atlantic right whales, *Eubalaena glacialis*. Females are born as calves and remain in the calf state for one year, then transition to the juvenile state, where they remain for eight years, after which, on becoming pregnant, they enter the adult stage. Maximum longevity is assumed to be 69 years. *S*_c,j_ is the probability of transitioning from calf to juvenile, *S*_j,j_ the probability of remaining in the juvenile state, *S*_j,a_ the probability of transitioning from juvenile to adult, and *S*_a,a_ the probability of remaining in the adult state; *f*_adlt_ is the probability of an adult whale giving birth to a calf. Solid black lines with arrows indicate the direction of transitions. R code for the figure is provided in electronic supplementary material 3.

We constructed a simple, three-stage population projection model for female right whales (figure 2). We selected the highest annual survival rates estimated for NARW from the Bayesian mark–resight analysis of photo-identifications published recently [6]. These were: calves: 0.96299; juveniles: 0.97507; and adult females: 0.97314, all from 2008. We assumed a juvenile stage duration of nine years [22,25,38,39], and a maximum longevity of 69 years [10]. Survival and transition probabilities for stages were calculated (using Equations 1 and 2 in [40]) (see R code in electronic supplementary material 3). We assumed a calving interval of four years, for the following reason. The mean calving interval for calving females from the NARW Catalog is 4.69 years (P Hamilton 2018, unpublished data). The mean observed calving intervals for SRW include 3.16 years for South Africa [39], 3.42 years for Argentina [41], 3.31 years for the Auckland Islands [27] and 3.3 years for Australia [25]. Rather than assume that NARW can reproduce as rapidly as SRW, we use four years as the approximate mid-point between the values for NARW and SRW.

The model was run in R 3.4.3 (R Core Team 2017), using the libraries *diagram* [42], *popbio* [43] and *popdemo* [44]. R code to run the model is included in electronic supplementary material 3.

## 3. Results

### 3.1. Calving over time

**Figure 3.**
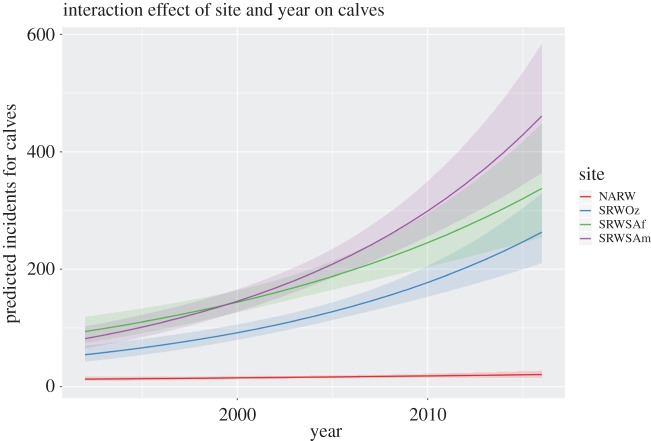
Right whale, *Eubalaena*, calf counts over time, 1992–2016. Model predicted slopes for all four sites (solid lines). Shaded areas are 95% confidence intervals of the slopes. Acronyms are as per figure 1. R code for the figure is provided in electronic supplementary material 2.

The slope of the GLM of calf counts over time (figure 3) for NARW was significantly different from all SRW Sites, none of which were significantly different from each other (table 1). The calf counts for NARW increased at 1.98% per year (s.e. 1.030), while SRW increased at 5.34% (s.e. 0.964), 6.58% (s.e. 0.861) and 7.21%/year (s.e. 0.845, table 2) for the South Africa, Southwest Australia and eastern South America populations, respectively.

**Table 1.** Results of the Negative Binomial Generalized Linear Model with log-link, including an interaction effect of population and Year. *p*-values were adjusted for multiple comparisons, using Benjamini & Hochberg's False Discovery Rate (in *phia::testInteractions*). NARW: North Atlantic right whale; SRWOz: Southern right whale, Southwest Australia; SRWSAf: Southern right whale, South Africa; SRWSAm: Southern right whale, eastern South America. R code for the GLM is provided in electronic supplementary material 2.

	value	d.f.	Chisq	Pr(>Chisq)
NARW-SRWOz	−0.046037	1	11.7675	0.0018082**
NARW-SRWSAf	−0.033561	1	5.6593	0.0347260*
NARW-SRWSAm	−0.052324	1	15.4249	0.0005151***
SRWOz-SRWSAf	0.012476	1	0.9317	0.4013015
SRWOz-SRWSAm	−0.006287	1	0.2717	0.6022247
SRWSAf-SRWSAm	−0.018763	1	2.1409	0.2151238
residuals		87		

*p < 0.05; **p < 0.01; ***p < 0.001.

**Table 2.** Back-transformed interaction means and standard errors for the Negative Binomial Generalized Linear Model (with log-link) of calf counts over time, 1992–2016. The point estimate (mean) is the average annual rate of increase, and the s.e. is the standard error of this estimate. Multiplication by 100 gives the annual percentage increase for each whale population over time. Acronyms are as per table 1. R code for the GLM is provided in electronic supplementary material 2.

site	mean	s.e.
NARW	0.0198	0.01030
SRWOz	0.0658	0.00861
SRWSAf	0.0534	0.00964
SRWSAm	0.0721	0.00845

### 3.2. Population projection models

Table 3 shows the matrix model used in the best NARW female survival analysis. The matrix presented in table 3 is the mathematical representation of the simplified, stage-structured model of the demography of female NARW, given the values listed above and calculating survival and transition probabilities for stages (using Equations 1 and 2 in [40]). Table 3's representation, using the same notation as figure 2, is:

$$\left[\begin{array}{ccc} 0 & 0 & F_{\mathrm{adlt}}\\ S_{c,j} & S_{j,j} & 0\\ 0 & S_{j,a} & S_{a,a} \end{array}\right].$$

**Table 3.** The matrix model used in the best NARW female survival analysis. R code calculating the matrix is provided in electronic supplementary material 3.

	calf	immature	adult
calf	0.00000	0.00000	0.1250
immature	0.96299	0.86368	0.0000
adult	0.00000	0.11139	0.9664

The intrinsic rate of increase for NARW is the dominant eigenvalue of this matrix [43] derived from this model which is 1.040 (i.e. 4% annual increase). Elasticity analysis [28] measures the proportional change in the intrinsic rate of increase driven by the proportional change of each of the matrix elements (i.e. survival of different life stages and fecundity). Given the stable stage distribution estimated from the matrix (the right eigenvector) and the reproductive value (the left eigenvector), elasticities can be calculated [28,43] (see electronic supplementary material 3 for R code). As elasticities sum to one [28], their meaning is straightforward to interpret. In this instance, elasticity analysis demonstrated that adult female mortality was proportionally the most important influence on asymptotic population growth rate (table 4), and accounted for about two-thirds of the change in NARW's intrinsic rate of increase.

**Table 4.** Results of elasticity analysis of the matrix used in the best NARW female survival analysis. R code for calculating the elasticity analysis is provided in electronic supplementary material 3.

	calf	immature	adult
calf	0.00000	0.00000	0.04740
immature	0.0474	0.23262	0.00000
adult	0.00000	0.04740	0.62517

**Figure 4.**
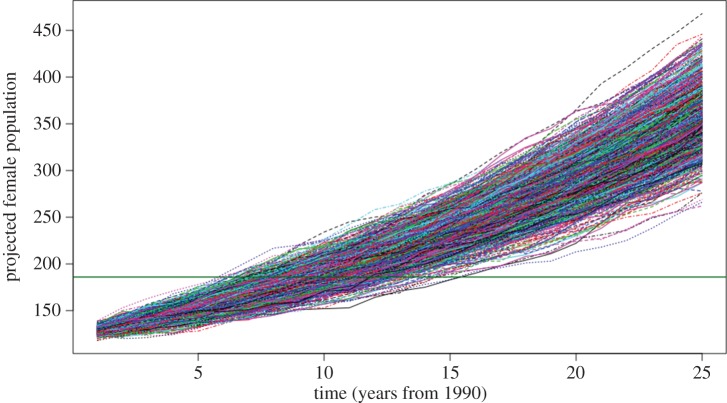
Projected population growth of female North Atlantic right whales. Projection uses the ‘best NARW female survival matrix' (table 3). Projection is for all female NARW: calves, juveniles and adults. The projection assumes a female population of NARW of 123 individuals in 1990 [6]. The proportions of each life-history stage for this starting population were calculated from the stable stage distribution of the matrix [28]. This estimated a starting population of 8 calves, 46 juveniles and 69 adults, and projects forward a further 25 years post-1990, to 2015. The horizontal green line demarcates 186 females, the estimated abundance of female NARW in 2015 [6]. R code for the figure is provided in electronic supplementary material 3.

In order to compare this intrinsic rate of increase with that observed, we ran 1000 stochastic projections of the matrix, from 1990 to 2015 (figure 4). First, we derived the proportions of each life-history stage from the stable stage distribution of the matrix [28]. From a starting population of 123 females in 1990 [6], this gave a 1990 population of eight calves, 46 juveniles and 69 adults (see electronic supplementary material 3). The median projected estimate 25 years later was 326 (95% quantiles: 266–393), female right whales of all stages (i.e. calves, juveniles and adults), compared to the observed 186 (95% credible intervals: 174–195, [6]).

## 4. Discussion

### 4.1. Why are NARW recovering more slowly than SRW?

The rate at which NARW calf counts have increased over the past 25 years (around 2% per year) is substantially less than that for the three SRW populations for which a comparable time series is available (between 5.3–7.2%/year). NARW calf production has increased slowly, with occasional periods of both relatively high and very low annual calf production (figure 1). Over the same time, the three SRW populations' patterns of recovery have been similar, with the numbers of calves born increasing relatively steadily, from dozens in the 1980s–1990s to hundreds recently.

To what extent is this difference in annual increase due to relatively greater ongoing anthropogenic impacts on NARW, rather than being ecologically intrinsic? NARW and SRW are similar animals, and were thought to be one species until molecular genetic analyses demonstrated otherwise [45]. The body condition of NARW, as estimated using visual health assessments, has been declining over recent years, and periods of reduced calving success coincide with periods during which all individuals of the species showed poorer overall health [46]. That female baleen whales forgo reproduction in response to poor body condition is well established [47]. In the southwest Australian population of SRW, longer and rounder females invested more energy into their calves than did smaller females in poorer condition [48]. Also, calves of larger, more rotund females gained more body volume over the three-month period at the calving ground than did calves of shorter, more thin females [48].

Stochasticity in NARW calving over time has been correlated with availability of *Calanus finmarchicus* [49]. Likewise, climate [50,51] and krill density [50] have been correlated with calf production of SRW off South America. Further studies comparing all SRW populations would be enlightening, but are beyond the scope of this paper. These findings help explain the stochasticity (figure 1) in calving time series, that is, the pattern of the residuals around the GLM's fit. What drives the slope of the calving trajectory is a different question, and the elasticity analysis of our matrix model demonstrates that the primary driver is adult female survival.

Furthermore, the current abundance of NARW, even at its recent maximum (just under 500 individuals, [6]) is substantially less than projections of their previous historical abundance (e.g. 9000–21 000, [52]). That zooplankton productivity and availability in the western North Atlantic has declined to the extent that food limitation is the sole reason for NARW's poor record of calving has not been demonstrated. In what other ways could adult female NARW be energy-limited?

The energy budget of any animal involves both energy intake and expenditure. Recently, a hitherto-ignored source of significant energy expenditure for NARW has been identified: entanglement in fishing gear [53]. Almost all individual NARW (83%) have been entangled at least once, and with many (59%) entangled two or more times [54]. The energetic demand from the drag associated with entanglement can be comparable to the cost of a one-way migration, and is sufficient to impact the likelihood that a female can successfully reproduce [53]. Entanglements can last from months to years, and recovery from entanglements can take similar time, so the time over which an entanglement episode affects a female NARW is also an issue [53]. For SRW, entanglement in fishing gear, when compared with NARW, is almost non-existent [55,56].

Adult female survival, rather than calving interval, is well established as having greatest influence on NARWs' intrinsic rate of increase [57]. Elasticity analysis of our model (table 4) shows adult female survival to be more than an order of magnitude more important than calving rate. Projections of our matrix model suggest that, had the survival of female NARW remained at the highest rates observed over the time series (0.975 for juveniles, 0.973 for adult females), and calving intervals been around four years, the species' numbers could have increased at 4%/year. Were that the case, our stochastic projections from that model indicate that there would have been almost twice the number of females in the species in 2015 as there actually were.

### 4.2. Implications for conservation

Thus, while some of the stochasticity in NARW calving may be environmental [48], the general slope of the recovery trajectory is driven by female mortality, particularly of adults. What drives female mortality in NARW? Over a period of 40 years, 1970–2009 inclusive, ∼80% (70 of 87) of NARW mortalities for which the cause of mortality is known (there were 122 mortalities identified overall), were anthropogenic ([58] numbers extracted from table 2 of that paper). This proportion is likely biased low as it does not differentiate calves of the year, which are more prone to natural mortality [55,59], from other age cohorts. With almost no observations from any other sources of mortality, a reasonable inference is that the vast majority of non-calf female NARW mortality is anthropogenic.

Similarly, most mortalities of SRW that have been observed are of calves of the year. The significant mortalities of calves of the year observed at Península Valdés, Argentina, in recent years [60] appear to be influenced by local environmental changes (including the behaviour of kelp gulls, *Larus dominicanus*, [61]). Most mortalities (for which an age class of the carcass could be determined) observed over 36 years (1963–1998 inclusive) off South Africa [56], and 57 years (1950–2006 inclusive) off southern Australia [55] were of calves of the year: 31 of 53 carcasses, and 16 of 28, respectively. The deaths of very few whales in either country were definitively anthropogenic: definitely eight, and possibly 16 of 55 SRW deaths off South Africa [56], and three of 28 off Australia [55].

Best *et al*. [56, p. 176], referring to SRW off South Africa, stated that ‘the current degree of anthropogenic mortality does not seem to pose a major conservation concern for this population’. While this statement is somewhat dated now, our analysis (figure 3) suggests it remains valid. Survival of adult female SRW off South Africa (data 1971–1998) was estimated at 0.986 (95% CI: 0.976–0.999), [62], or an annual mortality rate of 0.014, approximately half of the lowest annual mortality estimated (0.02686, in 2008) for adult female NARW. Note that, by using the highest estimates of female survivals from the Pace *et al*. [6] time series, we may still be overestimating what the mortality rate (i.e. underestimating survival) of female NARW would be if there were no anthropogenic mortality, and thereby underestimating NARW's possible intrinsic rate of increase. However, we chose our approach as we cannot rule out the possibility that there is at least some natural difference in the survival of NARW and SRW.

Our projections from the best NARW female survival model suggest there could have been around 326 female NARW in 2015 (and so at least 650 individuals in the species), if whales' survival had been consistently as good as the best observed in the 1990–2015 time series [6]. Had that been the case, the discovery of at least 17 dead right whales in 2017 would have been cause for alarm, but relative risk to the species would have been manageable. Instead, the recent detection of a decrease in NARW abundance since 2010 [6], coupled with the discoveries of mortalities in 2017, means that ‘the North Atlantic right whale is in deep trouble again’ [63].

For comparison, since 2003, there has been substantial mortality of SRW calves of the year on the calving grounds at Península Valdés, Argentina, with over 600 found dead to the end of 2013 [60,61]. Because the population of SRW in the western South Atlantic showed decades of substantial increase (figure 3) prior to these mortalities, the immediate risk to this population is far less than the current risk to NARW.

### 4.3. Caveats

We recognize that using counts of calves born each year could be biased, compared with estimates of absolute abundance for each right whale population. The three SRW populations are not surveyed on the foraging grounds, and only data from calving surveys are available. It is possible that some births are missed because of this. NARW are surveyed intensively throughout the year [10], so it is much less likely that births are missed. If the proportion of SRW births being missed increased over the time series, the SRW rates of increase over time would be biased low. If the proportion of SRW births missed remained constant over the time series, this would give an underestimate of the number of births, but not their rate of increase. If this bias exists, what it does not do is change the main finding of this comparison, that NARW are increasing more slowly than the SRW populations for which we have good calving time series.

Calf counts for right whales are collected in a similar manner at all four sites, so making direct comparisons with one analysis, as presented here, is superior to comparing rates of increase calculated using different methods [3]. Is it possible to compare a time series of calf counts with an independent time series of abundance estimates for right whales? Estimates of SRW abundance are extrapolated from the abundance of calves [23]. The only population for which there is a comparable, independent, published time series of mark–resight abundance estimates to compare with calf counts is NARW. A 26-year increase (1990–2015, [6]) from 270 [6] at the point estimate of increase from the calving index (i.e. 1.98%/year) gives an estimate of 450 whales [(1.0198^26^) × 270]. This estimate lies inside the 95% credible intervals of the abundance estimate for 2015 (444–471, [6]), demonstrating that the NARW calving time series is a reliable estimator of this species' trajectory over time.

A second caveat is that the values used in our population projection model for NARW population matrix were from observed data from the existing NARW time series. When using those values for the best NARW female survival we assume that the year with best survival values was one when anthropogenic impacts were minimized. In 2008, a year when the probability of photographically identifying each NARW was 94–95% (see [6], electronic supplementary material), there were no detected anthropogenic mortalities of NARW, and no mortalities for which the cause of death could not be determined [64]. The three mortalities detected in 2008 were all neonates that died of natural causes (A Henry 2018, personal communication). Although it is possible that our estimate of a maximum rate of increase for NARW is biased low, it is from a year when anthropogenic impacts of NARW survival seem at their lowest in the time series, and so the closest to what might be ‘natural’.

A third caveat is that some populations of SRW—those off southeastern Australian and Chile/Peru—are not recovering as rapidly as those off eastern South America, southern Africa, southwest Australia and the Auckland Islands/New Zealand. The reasons for these populations' relatively poor recovery are unclear but require assessment.

## 5. Conclusion

NARW have increased in abundance since 1990 at approximately 2% per year (including the decline in abundance observed since 2010), or approximately a third of the rate of increase demonstrated by at least three populations of their sister species, SRW. Projection models based on the best annual estimates of survival recorded for NARW suggest that they could increase at least 4% per year, over twice that observed. Elasticity analysis shows that adult female mortality is the key driver of the species' rate of change, and necropsy data over decades demonstrate that deaths of non-calf NARW are almost entirely due to anthropogenic causes.

Most studies of baleen whale conservation concentrate specifically on the one population of interest, and NARW are no exception to this norm. There are now multiple populations of some species of baleen whale for which times series of abundance are available [2]. A meta-analysis of recovery of humpback populations suggests differences in rates of recovery for those whales in the Northern and Southern Hemispheres [3]. Such comparisons can offer a broader context to our understanding of the recovery of particular species or populations. In this instance, by comparing NARW's recovery with multiple populations of SRW, we can make relatively strong inference that NARW recovery is unusually slow. Further, by coupling that analysis with our model projecting what NARW could do, given the best survival and calving rates that this species has demonstrated, we can address the question posed at the start of this paper: are these differences in recovery due to ongoing anthropogenic impacts or intrinsic ecological factors? Our results indicate that it is likely that NARW's maximum intrinsic rate of increase is less than that of SRW. However, we can conclude that anthropogenic mortality has limited the recovery of NARW, and that baseline conditions prior to their decline (post-2010) were already jeopardizing NARW recovery. Had NARW increased at the annual rate at which they are capable, the species' numbers would be almost double what they are now, and their current emergency would not be so dire.

## Supplementary Material

Supplement 1: survey data details

## Supplementary Material

Supplement 2: R code for GLMs

## Supplementary Material

Supplement 3: R code for population projection mdoel

## Supplementary Material

Supplement 4: calf count data over time (csv file)

## Data accessibility

The dataset and code supporting this article have been uploaded as part of the electronic supplementary material.
