# Supplementary material for: The recovery of North Atlantic right whales, Eubalaena glacialis, has been constrained by human-caused mortality
Source: R Soc Open Sci. 2018 Nov 7;5(11):180892. doi: 10.1098/rsos.180892 (PMC6281934; doi:10.1098/rsos.180892)
Supplement: Supplement 1: survey data details [file rsos180892supp1.docx]

**Corkeron et al. Supplementary Material 1.**

*Details of methods used to collect calf counts at each site over time.*

*NARW*. The number of calves born each calving season was extracted from the NARW Report Card, issued by the North Atlantic right whale Consortium each year and available at http://www.narwc.org. Neonatal calves are identified by association with their mother in the calving grounds off the south-east USA, from photographic identifications and genetic samples collected during aerial and small-vessel surveys (Hamilton et al. 2007). Surveys run from December to March (Brown et al. 2007). Occasionally (34 of 418), calves were first detected outside the heavily surveyed portion of the calving grounds, and a few are known to have been born elsewhere (e.g. Patrician et al. 2009). These are detected as new calves by their close association with a known adult female (presumed to be their mother), and confirmed by genetic matching of field samples (Hamilton et al. 2007) not observed with a calf previously in that calving season. NARW field sampling for calves is the most comprehensive of the data presented here. Further details of aerial surveys, and survey effort, for NARW in their calving grounds are available at Keller et al. (2006), Brown et al. (2007), and Gowan and Ortega-Ortiz (2014).

*SRW Southwest Australia*: Fixed-wing aerial surveys to count SRW calves off the western and central part of the southern coast of Australia have been ongoing since 1976 (Bannister et al. 2016). Between 1993 and 2006, one synoptic survey following the coastline both east-west and then reciprocally west-east, was run each year, along with two shorter survey flights. Since 2007, only the synoptic survey has been run each year. The survey is run in late-August / early-September at the time with the greatest likelihood that all females likely to calve that winter have done so, and have not yet left the calving grounds. Aerial surveys include counts and photo identification, on the assumption that all female-calf pairs in the survey area area detected. Unique calves are enumerated from the count data. Further details on field methods and analysis are available in Bannister et al. (2016). The data series analyzed here for Southwest Australia starts in 1993.

*SRW South Africa*: Surveys of SRW off the southern coast of South Africa have been ongoing since 1969, with helicopter surveys concentrating on photo-identification since 1979 (Best 1990). A unidirectional, synoptic survey is run to collect photo-identifications from which the number of individual calves born is enumerated. Further details on field methods and analysis are available in Best et al. (2001) and Findlay et al (2016). Calf counts for South Africa were available for 1992-2013.

*SRW eastern South America*: SRW calves are surveyed separately in Argentina and Brazil. There are no records to date of females photo-identified with calves in Argentina moving to Brazil within a calving season (or vice-versa), although females identified with calves off Argentina have been observed in other years calving off Brazil (Best et al. 1993, Rowntree et al. 2001, Danilewicz *et al.,* 2016).

In Argentina, fixed-wing aerial surveys have been conducted annually in the waters around Peninsula Valdez since 1971. Coastline surveys are cover the entire perimeter of the Peninsula at the time of peak whale abundance. During the surveys, the airplane circles over each group encountered while the photographer photo-identifies as many whales as possible and a note-taker records the number of female-calf pairs, lone adults and juveniles in a group (Payne 1986). The data used in this study come from the note-takers’s counts of female-calf pairs. Further details on field methods and analysis are available in Rowntree et al. (2001) and Rowntree et al (2013).

Off the coast of Brazil, aerial photo-identification surveys were conducted using fixed-wing aircraft (1987-1997) and helicopters thereafter (Groch et al. 2005). For Brazil, data from 1995, 1996 and 2014 were unavailable, as surveys were not run due to funding shortfalls. Further details on field methods and analysis are available in Groch et al. (2005) and Seyboth et al. (2016).

Calf counts enumerated separately in each of Brazil and Argentina were summed to derive an estimate of calving for “eastern South America”.

**References**

Bannister, JL, Hammond, PS and Double MC et al. 2016. Population trend in right whales off southern Australia 1993-2015. International Whaling Commission document SC/66b/BRG/09

Best, PB 1990. Trends in the inshore right whale population off South Africa, 1969–1987. Marine Mammal Science, 6: 93–108. doi:10.1111/j.1748-7692.1990.tb00232.x

Best PB, Brandão A and Butterworth DS 2001. Demographic parameters of southern right whales off South Africa. Journal of Cetacean Research and Management (Special Issue 2) 161-169.

Best PB, Payne, R, Rowntree, V, Palazzo, JT and Both MDC 1993. Long-range movements of South Atlantic right whales, *Eubalaena australis*. Marine Mammal Science 9:227–234. doi:10.1111/j.1748-7692.1993.tb00451.x

Brown, MW, Kraus, SD, Slay, CK, and Garrison, LP 2007. Surveying for discovery, science and management. Pp 105-137 in Kraus, SD, and Rolland RR (eds). The Urban Whale. Harvard University Press. Cambridge MA USA.

Danilewicz D, Moreno IB, Tavares M, and Sucunza F 2016. Southern right whales (*Eubalaena australis*) off Torres, Brazil: group characteristics, movements, and insights into the role of the Brazilian-Uruguayan wintering ground. *Mammalia.* Doi: 10.1515/mammalia-2015-0096

Findlay, KP, Thornton, MT, Peters, IT and Best, PB 2016. Report of the 2014 Mammal Research Institute Whale Unit Southern right whale survey, Nature’s Valley to Muizenberg, South Africa. International Whaling Commission document SC/66b/BRG/4

Groch, KR Palazzo, JT Jr, Flores, PAC, Adler, FR, Fabian ME 2005. Recent rapid increases in the right whale (*Eubalaena australis*) population off southern Brazil. Latin American Journal of Aquatic Mammals. 4:41-47 <http://www.lajamjournal.org/lajam/index.php/lajam/article/view/212>.

Hamilton PK, Knowlton AR, and Marx MK 2007. Right whales tell their own stories: the photo-identification *Catalog*. Pp75-104 in Kraus, SD, and Rolland RR (eds). The Urban Whale. Harvard University Press. Cambridge MA USA.

Gowan TA, Ortega-Ortiz JG 2014. Wintering Habitat Model for the North Atlantic Right Whale (Eubalaena glacialis) in the Southeastern United States. PLoS ONE 9(4): e95126. https://doi.org/10.1371/journal.pone.0095126

Keller, CA, Ward-Geiger, LI, Brooks, WB, Slay, CK, Taylor, CR and Zoodsma, BJ 2006. North Atlantic right whale distribution in relation to sea-surface temperature in the southeastern United States calving grounds. Marine Mammal Science, 22:426–445. doi:10.1111/j.1748-7692.2006.00033.x

Patrician, MR, Biedron, IS, Esch, HC, Wenzel, FW, Cooper, LA, Hamilton, PK, Glass, AH and Baumgartner, MF 2009. Evidence of a North Atlantic right whale calf (Eubalaena glacialis) born in northeastern U.S. waters. Marine Mammal Science, 25:462–477. doi:10.1111/j.1748-7692.2008.00261.x

Payne R 1986. Long term behavioral studies of the Southern right whale (Eubalaena australis). Reports of the International Whaling Commission. Species Issue 10:161-167.

Rowntree V, Payne RS and Schell DM 2001. Changing patterns of habitat use by southern right whales (*Eubalaena australis*) on their nursery ground at Península Valdés, Argentina, and in their long-range movements. Journal of Cetacean Research and Management (Special Issue) 2:133–143.

Rowntree VJ, Uhart MM, Sironi M, Chirife A and others (2013) Unexplained recurring high mortality of southern right whale *Eubalaena australis* calves at Penïnsula Valdïs, Argentina. Marine Ecology Progress Series 493:275-289. <https://doi.org/10.3354/meps10506>

Seyboth E, Groch, KR, Dalla Rosa L, Reid, K, Flores, PAC, Secchi, ER. 2016. Southern Right Whale (*Eubalaena australis*) reproductive success is influenced by krill (*Euphausia superba)* density and climate. Scientific Reports 6:28205 http://dx.doi.org/10.1038/srep28205
